# Influence of Flank Wear on the Microstructure Characteristics of the GH4169 Metamorphic Layer under High-Pressure Cooling

**DOI:** 10.3390/ma16082944

**Published:** 2023-04-07

**Authors:** Min Wei, Mingyang Wu, Jiamiao Xu, Yaonan Cheng

**Affiliations:** Key Laboratory of Advanced Manufacturing and Intelligent Technology, Ministry of Education, Harbin University of Science and Technology, Harbin 150080, China; minwei0126@163.com (M.W.); 18846047219@163.com (J.X.); yaonancheng@163.com (Y.C.)

**Keywords:** high-pressure cooling, metamorphic layer, microstructure, flank wear

## Abstract

Since the flank has an important influence on the surface of a workpiece, and as microstructure flaws of the surface metamorphic layer are a key factor that affects the service performance of a part, this work studied the influence of flank wear on the microstructure characteristics of the metamorphic layer under the conditions of high-pressure cooling. First, Third Wave AdvantEdge was used to create a simulation model of cutting GH4169 using tools with different flank wears under high-pressure cooling. The simulation findings emphasized the impact of flank wear width (VB) on the cutting force, cutting temperature, plastic strain, and strain rate. Second, an experimental platform was established for cutting GH4169 under high-pressure cooling, and the cutting force during the machining process was recorded in real time and compared with the simulation results. Finally, an optical microscope was used to observe the metallographic structure of the GH4169 workpiece section. The microstructure characteristics of the workpiece were analyzed using a scanning electron microscope (SEM) and electron backscattered diffraction (EBSD). It was discovered that, as the flank wear width increased, so did the cutting force, cutting temperature, plastic strain, strain rate, and plastic deformation depth. The relative error between the simulation results of the cutting force and the experimental results was within 15%. At the same time, near the surface of the workpiece, there was a metamorphic layer with fuzzy grain boundaries and refined grain. With an increase in flank wear width, the thickness of the metamorphic layer increased from 4.5 μm to 8.7 μm and the grain refinement intensified. The high strain rate promoted recrystallization, which caused an increase in the average grain boundary misorientation and high-angle grain boundaries, as well as a reduction in twin boundaries.

## 1. Introduction

GH4169 is a superalloy material that has found widespread use in the fields of aeronautics, ships, and other industries. In complicated and severe working environments, it demonstrates excellent fatigue resistance, fracture toughness, and high-temperature strength [1]. This working environment, however, places additional demands on the performance of superalloy components. Due to the GH4169 superalloy’s high strength and limited plasticity, considerable thermal-mechanical coupling and plastic deformation occur during the cutting process, resulting in metamorphic layers on the machined surface with characteristics distinct from the material matrix [2,3]. The fatigue failure of components is partially caused by microstructure faults in the metamorphic layer on the cutting surface of the workpiece, according to a service fault analysis of the hot-end parts of an aero-engine [4]. Wu et al. [5] combined simulation with experiments to explore the effects of cutting speed, feed rate, and cooling pressure on the thickness of the metamorphic layer, and residual stress on the workpiece surface under high-pressure cooling. The results indicated that an increase in cutting speed and feed rate enhanced the residual stress and promoted formation of the metamorphic layer, while an increase in coolant pressure weakened the residual stress and reduced the thickness of the metamorphic layer. Schlauer et al. [6] investigated the distribution of residual stress in a workpiece cross section while cutting Inconel 718 at various cutting speeds and feed rates. Transmission electron microscopy revealed that grain slide occurred on the workpiece surface, aggravating the degree of refining. Zhang et al. [7] researched the effects of cutting speed and cutting depth on the stress, temperature, and dislocation density of the cutting layer, based on a simulation analysis, with nano-cut GH4169 as the experimental object. Nasralla et al. [8] established a 3D simulation model of cutting Inconel 718 and verified through experiments that the cutting speed had a significant impact on the residual stress and microhardness, while feed had a substantial influence on the surface roughness. Gürbüz et al. [9] evaluated the cutting performance of cryogenically treated and untreated tools when machining Inconel 718, and they discovered that the former produced a smoother surface. Peng et al. [10] built a prediction model of residual stress for an Inconel 718 turning surface based on finite element simulation and experiment results. Ruzzi et al. [11] reported that the roughness of the cutting surface achieved by grinding nickel-based superalloy with a GC grinding wheel was lower than that obtained by grinding with a WA grinding wheel. Le et al. [12] pointed out that low-temperature machining outperformed dry cutting, minimal lubrication, and nanofluids in managing workpiece surface flaws. Frifita et al. [13] established an optimization model of cutting parameters of an Inconel 718 superalloy, aiming at the minimizing the surface roughness and cutting force. Mickael et al. [14] observed recrystallization grains, twins, and high-density dislocations in the metamorphic layer of a Ti-6Al-4V machined surface.

Researchers proposed the high-pressure cooling cutting technique to optimize the processability of GH4169, which offers technological assistance for cutting difficult-to-cut materials. Lopez D L et al. [15] found in experiments that, when cutting titanium alloy with a coolant pressure of 11 Mpa, the chips were more prone to curling and breakage, and the cutting edge of the tool showed little fracturing or plastic deformation. A. Suárez et al. [16] cut Haynes 282 superalloy under conventional and high-pressure cooling, which proved that the high-pressure cooling reduced the average feed force by 20%, but the flank and notch wear did not obviously improve. Meanwhile, another study [17] pointed out that high-pressure cooling reduced the flank wear by more than 30% and the temperature by about 50%, as well as also affecting the notch wear. R. Polvorosa et al. [18] believed that the grain size of a material is the key to tool wear and that there is no necessary correlation between high-pressure cooling and notch formation for 718 alloy, but for a large-grain Waspaloy, high-pressure cooling can alleviate notch wear. Zydrunas et al. [19] conducted a cutting experiment on Inconel 718 with SiAlON ceramic tools, which showed that the tool life for high-pressure cooling was longer than that during conventional cutting. Yusuf K et al. [20] studied the effects of low temperature, MQL, and high-pressure cooling on the chip formation of Ti-5553 alloy, and the findings indicated that high-pressure cooling shortened the tool-chip contact length. These studies proved that high-pressure cooling technology has a positive effect on machining and provided theoretical guidance for this work.

Tool wear has long been a pressing issue in the mechanical professions. The continual wear of a tool becomes the key variable during the cutting process, and the friction and extrusion between the tool flank and the cutting surface cause the flank wear to have a noticeable impact on the microstructure of the metamorphic layer. Oliveira A.R.F. et al. [21] studied the surface integrity of Inconel 718 alloy end milling with cemented carbide tools, and found that flank wear reduced the microhardness of the workpiece surface and produced residual tensile stress, which further affected the surface roughness of the workpiece. Kong et al. [22] reported that with a higher flank wear, there was a deeper deformation zone of the metallographic structure on the surface and a higher degree of grain distortion and tensile plastic deformation. Zhang et al. [23] established an evaluation system of flank wear considering surface roughness, which can guide and control the influence of flank wear on machined surface roughness. Xie et al. [24] investigated the influence of tool wear on the surface integrity of GH4169 during high-speed cutting. The experimental results indicated that intensified tool wear caused the increase of surface microhardness and roughness. When milling Ti60, Yao et al. [25] found that, with the increase of flank wear, a twisted and slid microstructure was visible on the material surface and the plastic deformation layer became thicker. Gaurav B et al. [26] discussed the influence of tool wear and cutting speed on the thickness of the white layer through a regression linear analysis. With the deterioration of tool wear, it is necessary to control the thickness of the white layer with a smaller cutting speed.

In summary, although domestic and international scholars have conducted extensive research on the machined surface of superalloys, they primarily studied the thickness, residual stress, and roughness of the metamorphic layer on the machined surface, as a result of the cutting parameters, machining methods, and so on, while little research has been conducted on the microstructural characteristics of the metamorphic layer of the machined surface as a result of flank wear under high-pressure cooling. However, this research is of great significance for improving the serviceability of superalloy parts. On the premise of turning GH4169 with a PCBN tool under high-pressure cooling, first, Third Wave AdvantEdge was used to simulate and analyze the cutting force, cutting temperature, plastic strain, and strain rate of the cutting surface while machining GH4169 with a coolant pressure of 50 bar. Second, a GH4169 turning experiment under high pressure cooling was carried out, and the cutting force was measured and compared with the simulation findings. Finally, the metallographic structure of the GH4169 workpiece section was observed utilizing an optical microscope; the thickness, grain size, and grain boundary misorientation of the metamorphic layer was explored using SEM and EBSD; and the influence of the flank wear width on the microstructure characteristics of the metamorphic layer is discussed. This work will provide a theoretical foundation for producing high-quality machined surfaces and for enhancing component service life.

## 2. Simulation Analysis of Cutting GH4169 with Different Flank Wear Tools

### 2.1. Establish Cutting Simulation Model

**Figure 1:**
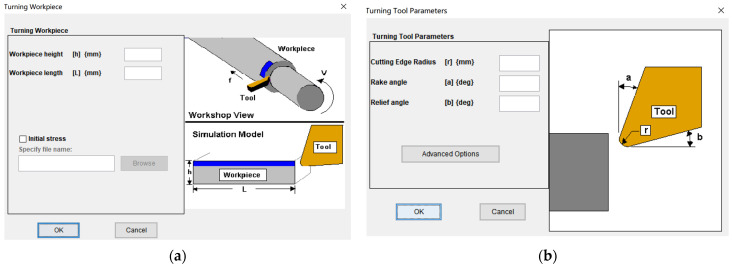
Workpiece–tool parameter settings: (**a**) standard workpiece; (**b**) standard tool.

**Figure 2:**
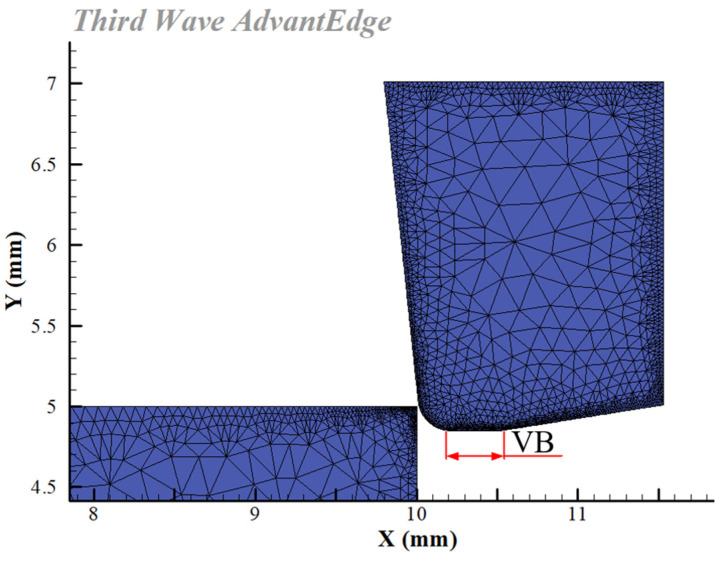
Two-dimensional simulation model of cutting GH4169.

Third Wave AdvantEdge simulates the GH4169 cutting process under high-pressure cooling, where the material constitutive model is PowerLaw, the chip separation criterion is physical separation, the tool–workpiece–chip friction model is Coulomb friction, and the tool wear model is Usui. As the tool clearance angle progressively smooths out with wear, the wear plane width equals the flank wear width (VB). Based on a user-defined tool module for simulation software, this article simulated tools with varied flank wear widths in finite element modeling. The standard model of cutting tool and workpiece was selected, as shown in Figure 1. The workpiece material was GH4169, the tool was PCBN. The adaptive mesh division method was used to divide the mesh of tool and workpiece. Other parameter settings are shown in Table 1. Figure 2 depicts the 2D simulation model for cutting GH4169 when the VB was 0.3 mm.

### 2.2. Simulation Results

#### 2.2.1. Analysis of Cutting Force Simulation Results

**Figure 3:**
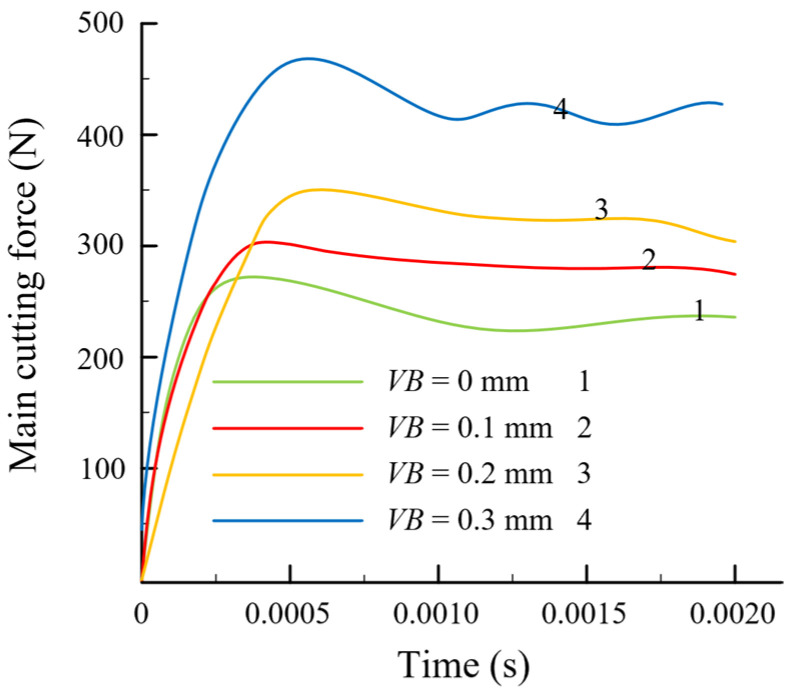
Simulation results for cutting force.

According to the cutting force simulation results shown in Figure 3, the cutting force immediately climbed to its maximum value at the beginning of processing and then progressively stabilized as the cutting time progressed. The cutting force increased as the flank wear width increased. The primary cause of this phenomena is that flank wear decreases the tool clearance angle, expands the contact area between the flank and the workpiece surface, and boosts the cutting force, owing to the growing friction in the contact region. The cutting force results proved similar to those reported in [27].

#### 2.2.2. Analysis of Cutting Temperature Simulation Results

**Figure 4:**
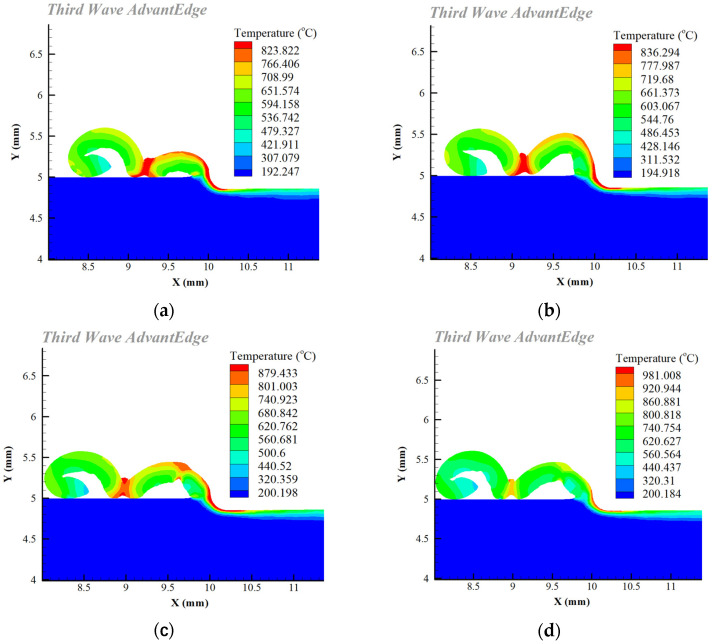
Simulation results for cutting temperature: (**a**) VB = 0 mm; (**b**) VB = 0.1 mm; (**c**) VB = 0.2 mm; (**d**) VB = 0.3 mm.

As shown in Figure 4, the simulation results for cutting temperature revealed that the VB was 0 to 0.2 mm, the tool cutting edge wear was modest, and the friction with the workpiece was not severe, so the cutting temperature went up slowly. When the VB was between 0.2 and 0.3 mm, the tool wear accelerated and the contact area between the tool and the workpiece expanded, leading to friction heat generation. Furthermore, the workpiece material’s elastic–plastic deformation intensified, and the work performed by elastic recovery increased, resulting in a significant increase in the cutting temperature.

#### 2.2.3. Simulation Results for the Strain Field

**Figure 5:**
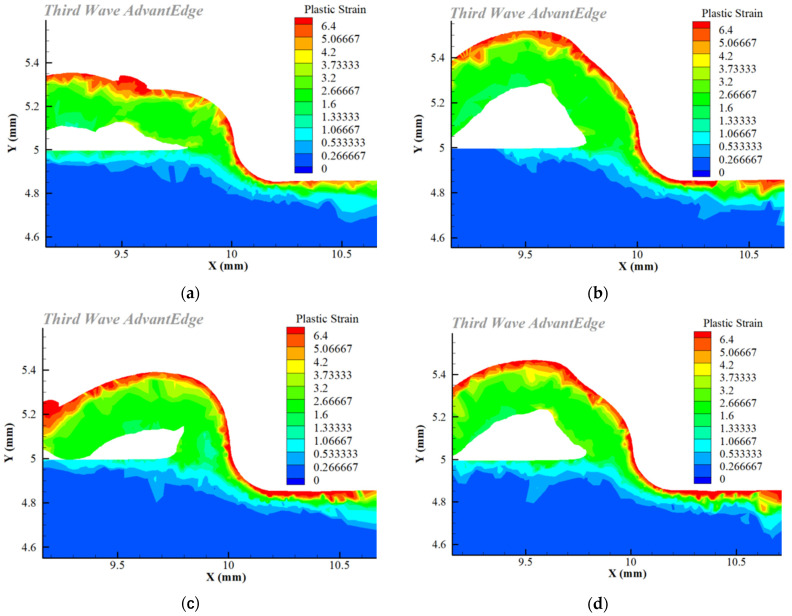
Simulation results for plastic strain: (**a**) VB = 0 mm; (**b**) VB = 0.1 mm; (**c**) VB = 0.2 mm; (**d**) VB = 0.3 mm.

It is shown in Figure 5 that the maximum plastic strain existed in the surface layer of the workpiece, and the plastic strain gradually weakened in the depth direction.

**Figure 6:**
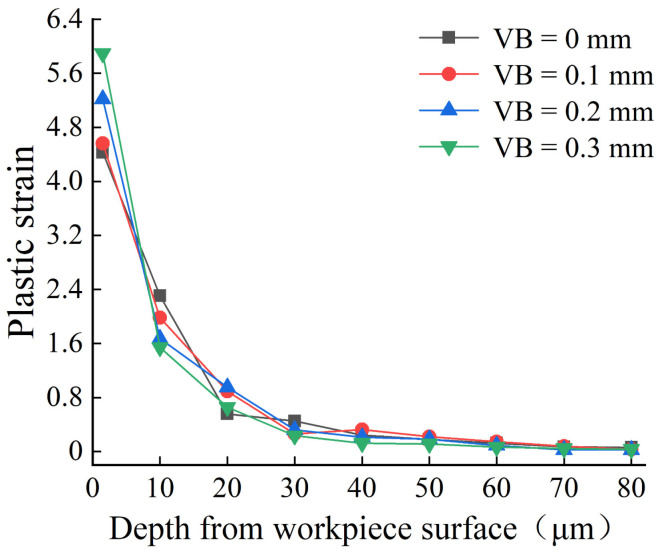
Plastic strain through the depth of workpiece surface.

**Figure 7:**
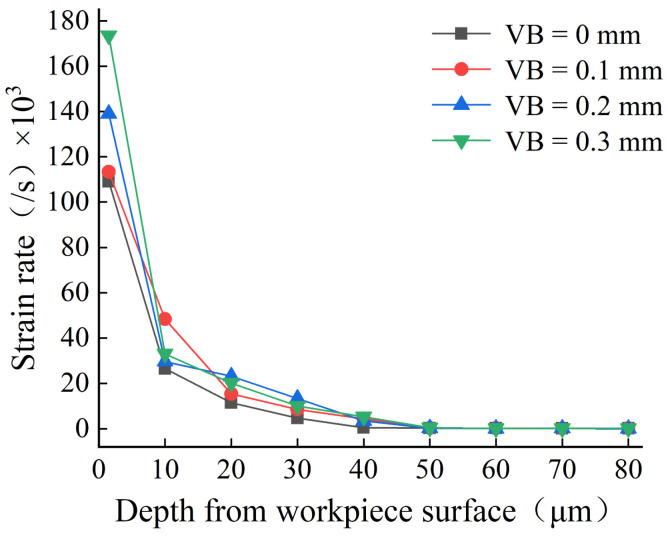
Strain rate in the depth direction of the workpiece surface.

After cutting with various flank wear tools, the plastic strain and strain rate of the workpiece surface were recorded by taking a measurement every 10 m in the depth direction of the workpiece surface. Figure 6 and Figure 7 show that as the flank wear width increased, the plastic strain and strain rate increases, and the strain impact progressively grew. This is because increasing the flank wear width worsens the friction in the contact region and increases the stress, resulting in a higher plastic strain and strain rate, as well as an increase in the degree of plastic deformation. Zhang [28] and Feng [29] also obtained the same conclusion.

## 3. Experiment on Cutting GH4169 with a PCBN Tool under High-Pressure Cooling

### 3.1. Experiment Conditions

The workpiece material was a superalloy GH4169 bar after a solution aging treatment. Its size was ϕ92 × 350 mm, and its chemical composition and physical properties are shown in Table 2 and Table 3.

The cutter was a PCBN blade produced by Secotools, the model was CNGA120408E25-L1-U CBN170, and the geometric parameters of the PCBN inserts are shown in Table 4. The tool holder model was DCLNR2525X12JETI. After the tool and the tool holder had been installed, the cutting angle were established, with the rake angle being −6°, the relief angle being 6°, the tool cutting edge angle being 95°, and the inclination angle being −6°.

The equipment used was a CKA6150 CNC lathe produced by the Dalian Machine Tool Works, and the cooling system was a SOONWELL external high-pressure pump and the MS590XT emulsion. During the turning process, the parameters of injection diameter, distance, and coolant angle remained constant.

### 3.2. Experiment Schemes

Table 5 illustrates the combination of experiment parameters, aiming at exploring the influence of flank wear width on the cutting force during cutting of GH4169 under high-pressure cooling conditions and obtaining corresponding workpiece samples.

**Figure 8:**
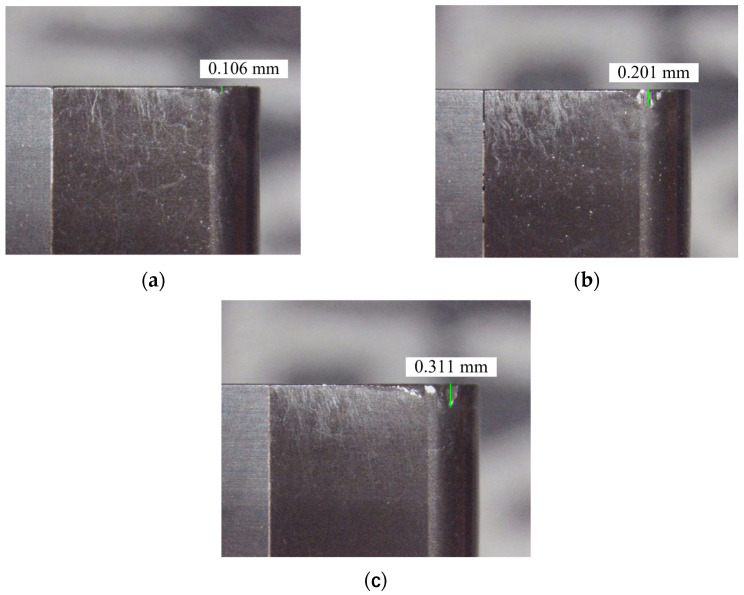
Pre-ground PCBN tool: (**a**) VB = 0.1 mm; (**b**) VB = 0.2 mm; (**c**) VB = 0.3 mm.

When studying the effect of the various flank wear widths, the PCBN tool was pre-ground, and the flank wear width was monitored using an industrial camera, until the wear loss reached the experimental default, and then the cutting experiment with relevant parameters was carried out. Figure 8 depicts a pre-ground PCBN tool.

**Figure 9:**
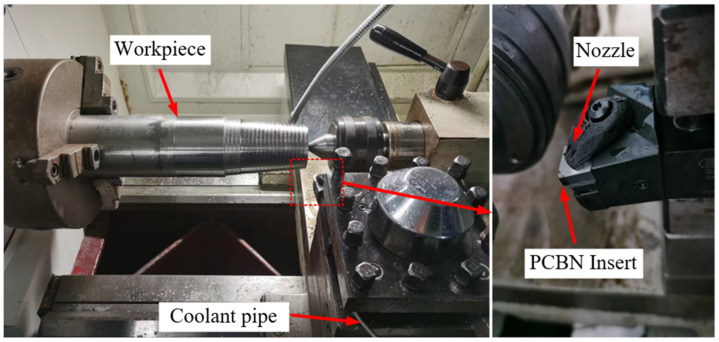
Experimental device for cutting GH4169 under high-pressure cooling.

To eliminate the impact of oxides or impurities on the experimental findings, the superalloy GH4169 bar surface layer was turned prior to the experiment, and the workpiece was then turned into a stepped bar, according to each set of experimental parameters. Simultaneously, a measuring system consisting of a Kistler 9139AA piezoelectric dynamometer, Kistler 5167A charge amplifier, and DynoWare data acquisition software was used to measure the cutting force during the cutting process, as shown in Figure 9, which shows the experimental device for cutting GH4169 under high-pressure cooling.

After the turning experiment was completed, a workpiece sample with a size of 10 × 5 × 5 mm was cut on each step of the bar through wire cutting, and the workpiece was inlaid, ground, polished, corroded, and cleaned. Finally, metallographic samples required for the SEM and EBSD were obtained, paving the way for further investigation of the microstructural characteristics of the deteriorated layer of the cutting surface.

### 3.3. Experiment Results

**Figure 10:**
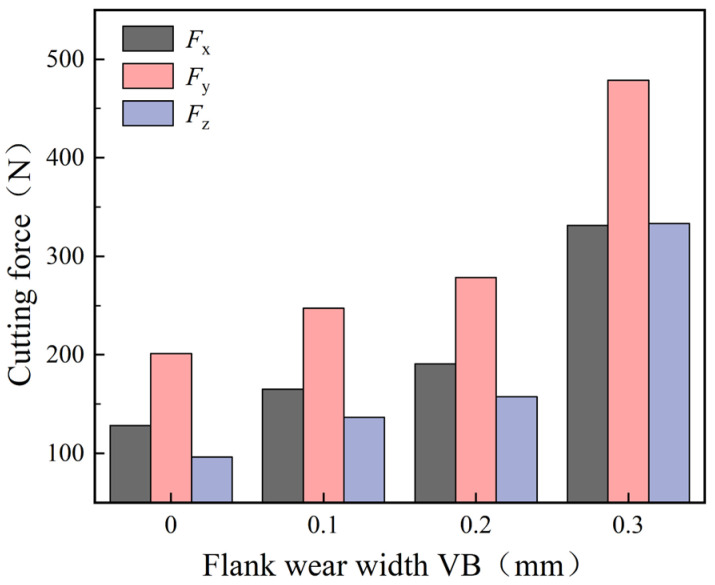
Experimental results for cutting force.

The experimental cutting force data in Figure 10 clearly indicate the effect of flank wear on the axial force (*F_x_*), tangential force (*F_y_*), and radial force (*F_z_*) during high-pressure cooling cutting.

As can be seen from Figure 10, when the VB was between 0 and 0.3 mm, the total cutting force gradually increased, as the flank wear intensified. When the VB climbed from 0 to 0.2 mm, the tool was in the initial and normal wear stages, and the cutting force increased slowly. However, when the VB increased from 0.2 to 0.3 mm, the tool entered a stage of sharp wear and the cutting force increased greatly. Compared with the tool without wear, when VB was 0.3 mm, the *F_x_* increased by 203 N, the tangential *F_y_* increased by 277 N, and the radial *F_z_* increased by 237 N. Therefore, it can be seen that the flank wear had the greatest influence on the *F_y_* and the smallest influence on the *F_x_*. Thus, the overall change trend of cutting force was in good agreement with the simulation results, indicating that the simulation results were highly credible.

The experimental and simulation results of cutting force are listed in Table 6. The relative error between them is within 15%, and it can be considered that the overall change trend of the cutting force was in good agreement with the simulation results, which shows that the simulation results had good credibility.

## 4. Influence of Flank Wear on the Metamorphic Layer Thickness

**Figure 11:**
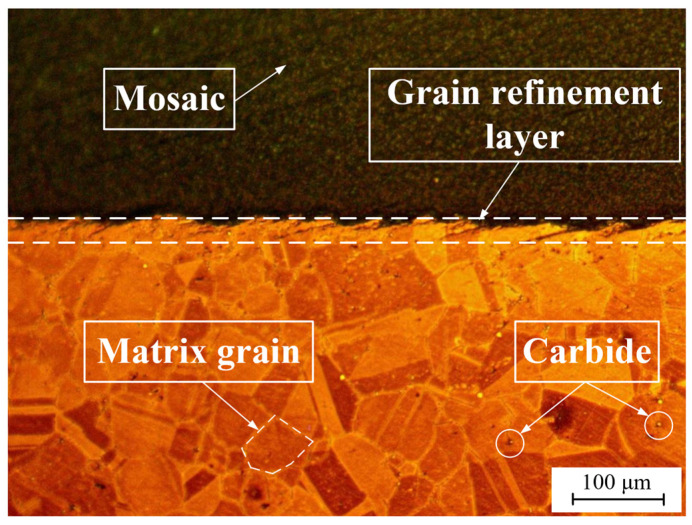
Metallographic structure of the GH4169 workpiece cross-section.

**Figure 12:**
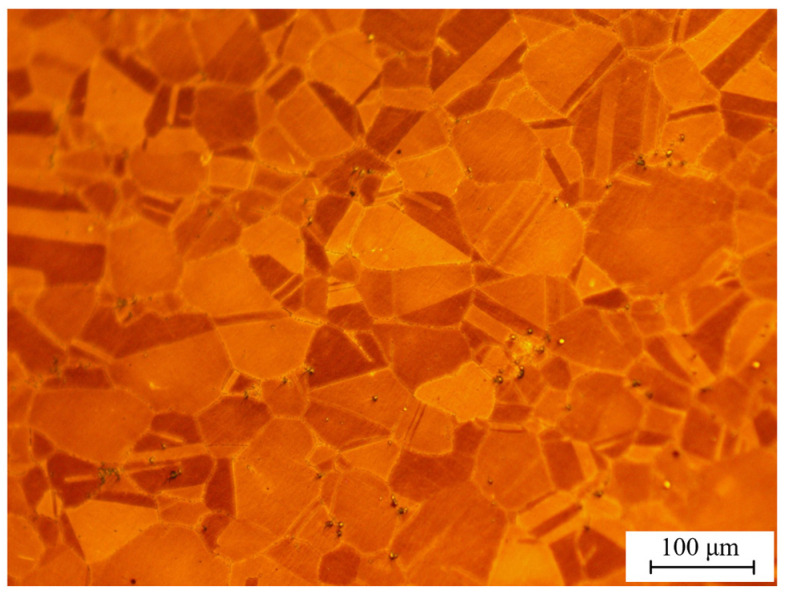
Initial microstructure of the GH4169.

Figure 11 depicts the metallographic structure of a cross-section of the superalloy GH4169. According to the phase diagram, the matrix material of GH4169 had a uniform grain size, distinct grain boundaries, and a handful of carbides, which is similar to the original microstructure of GH4169 shown in Figure 12. Nevertheless, grain refinement occurred on the workpiece surface, making the grain boundary harder to distinguish. The grain was fragmented and deformed, with a severely deformed plastic structure. Metamorphic layer is the name given to this area.

**Figure 13:**
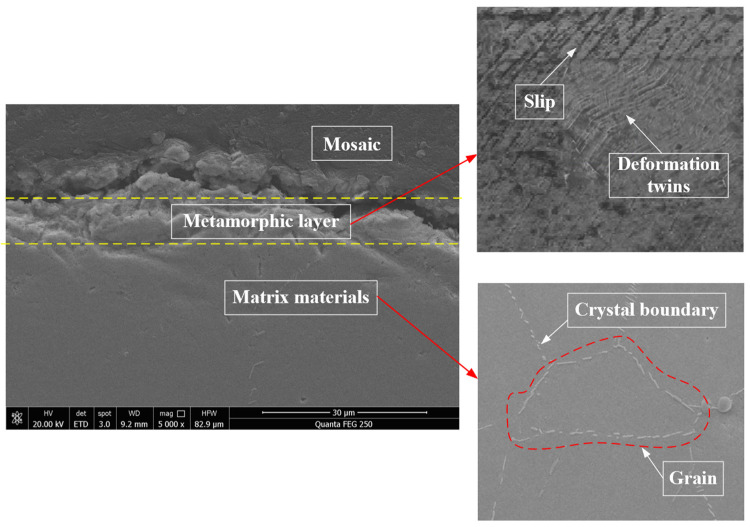
The cross-sectional microstructure of the GH4169 workpiece.

The microstructure of the cross-section of the GH4169 workpiece is illustrated in Figure 13. The metamorphic layer of a certain thickness was clearly distinguished from the matrix material using SEM. The local enlargement of the metamorphic layer and the matrix material revealed that the metamorphic layer grains were twisted and deformed along the cutting direction, which indicated that plastic deformation occurred during cutting. At this time, a significant number of dislocation blocks prevented the dislocations from moving further, resulting in deformation twins as the internal stress progressively built to the critical cutting stress. The matrix material, on the other hand, had regular grains and distinct grain boundaries. Yao et al. [25] also found grain slip and deformation in the microstructure of a workpiece, presumably related to mechanical extrusion and thermal softening effects.

**Figure 14:**
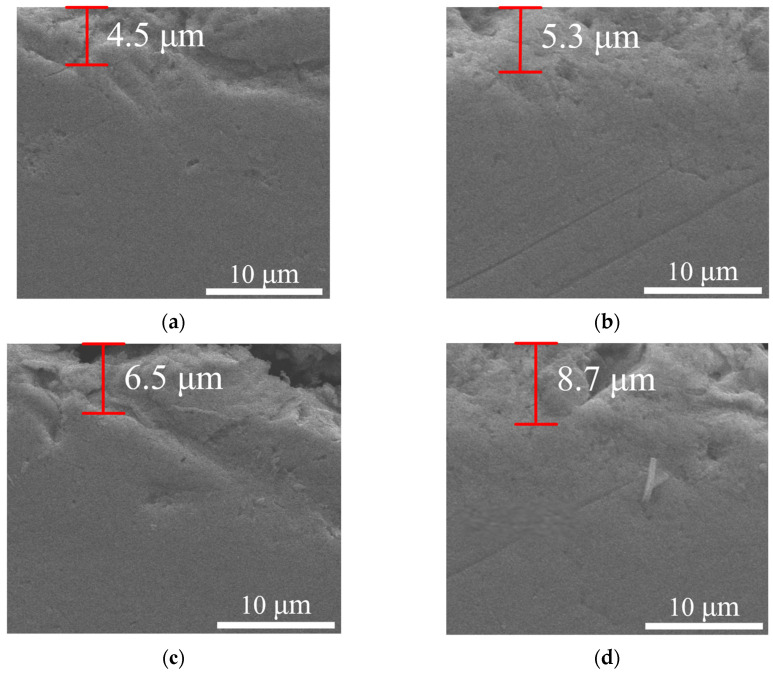
Influence of flank wear on metamorphic layer thickness under high-pressure cooling: (**a**) VB = 0 mm; (**b**) VB = 0.1 mm; (**c**) VB = 0.2 mm; (**d**) VB = 0.3 mm.

When tool machining with various flank wears, the thickness of the metamorphic layer was measured. The equivalent metamorphic layer thickness was 4.5 μm, 5.3 μm, 6.5 μm, and 8.7 μm when the flank wear width was 0 mm, 0.1 mm, 0.2 mm, and 0.3 mm. From Figure 14, we can draw the conclusion that the thickness of the metamorphic layer went up as the flank wear width increased. This phenomenon was primarily caused by a significant thermal-mechanical coupling effect when the flank wear width was too large, which intensified the plastic deformation of the material cutting layer and promoted grain refinement. Moreover, the degree of influence and depth of stress and strain increased, which was beneficial for the formation of the metamorphic layer, thus the metamorphic layer thickness increased. A. Attanasio [30] suggested that the thickness of the metamorphic layer increased because the worn tools generated higher temperatures on the machined surface and a deeper heat-affected zone, due to the higher frictional contact effect.

## 5. Influence of Flank Wear on the Grain Morphology and Grain Size

**Figure 15:**
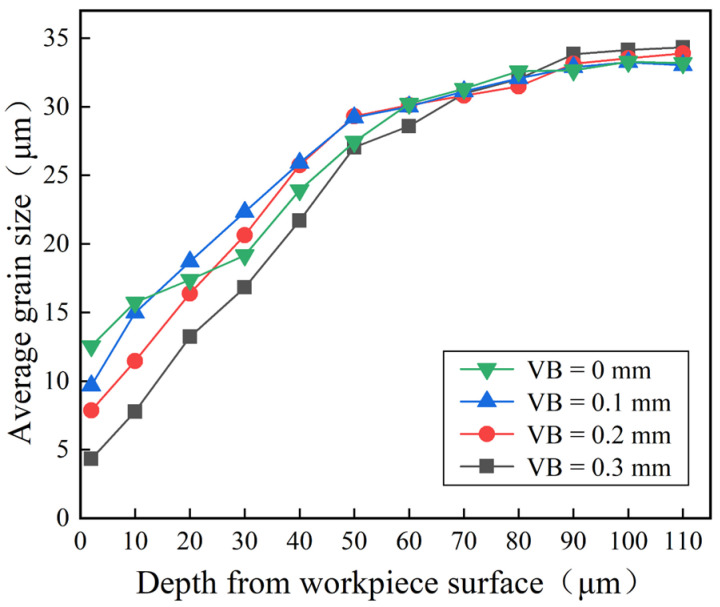
Statistics of the average grain size distribution in the depth direction in the cross-section of the GH4169 workpiece.

The EBSD technique was used to quantitatively analyze the grain size and grain boundary misorientations of the cutting metamorphic layer. The average grain size was determined by selecting locations in the workpiece’s surface layer at 10 μm intervals in the depth direction. According to Figure 15, the average grain size exhibited a gradient increase in the depth direction and progressively approached the grain size of the initial structure of GH4169. In other words, the grain became denser as it moved closer to the machined surface. Ren [31] reported a similar findings for the grain size distribution in the depth direction.

**Figure 16:**
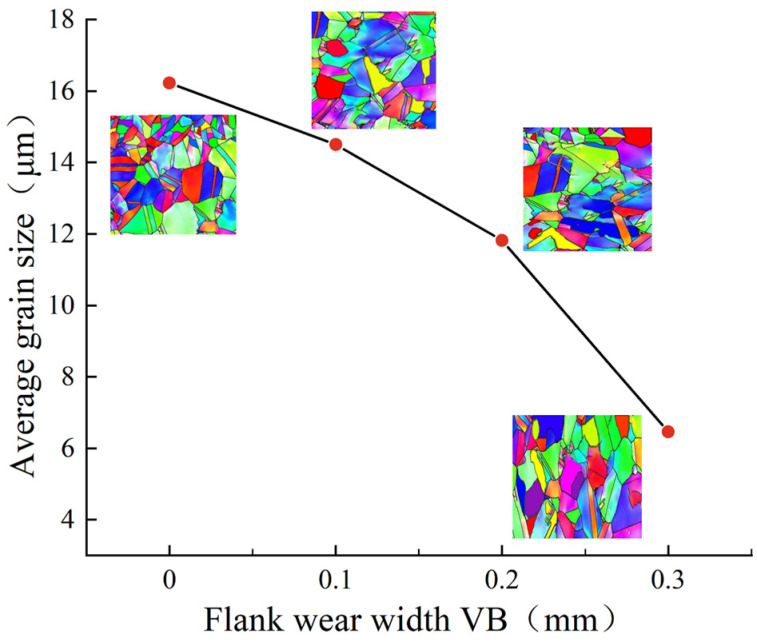
Statistics of the grain morphology and average size in the GH4169 cutting metamorphic layer under different flank wears.

Figure 16 shows the statistical results of the grain morphology and average grain size in the cutting metamorphic layer under different flank wears. When the flank wear width was 0 mm, 0.1 mm, 0.2 mm, and 0.3 mm, the average grain size in the cutting metamorphic layer was 16.22 μm, 14.50 μm, 11.82 μm, and 6.46 μm, respectively. It was concluded that, with the increase of flank wear width, the average grain size in the cutting metamorphic layer decreased gradually. The reason for this was that the cutting force, cutting temperature, and strain rate were greatly increased due to the intensified flank wear, the tool flank and the cutting surface were violently rubbed, and the workpiece surface was severely plastically deformed under the action of thermal-mechanical coupling, while the dynamic recrystallization was accelerated with the increase of temperature, which led to the intensification of grain refinement [31]. At the same time, it was found that when the VB was 0 to 0.2 mm, the flank mainly had abrasive wear and boundary wear, and the wear degree was light and the influence of tool wear on the workpiece surface was small, which made the change in the degree of grain refinement relatively slow. When the VB was 0.2 to 0.3 mm, the phenomenon of edge collapse and damage appeared on the tool flank and the effect of mechanical load on the workpiece surface was enhanced, resulting in a denser grain size.

**Figure 17:**
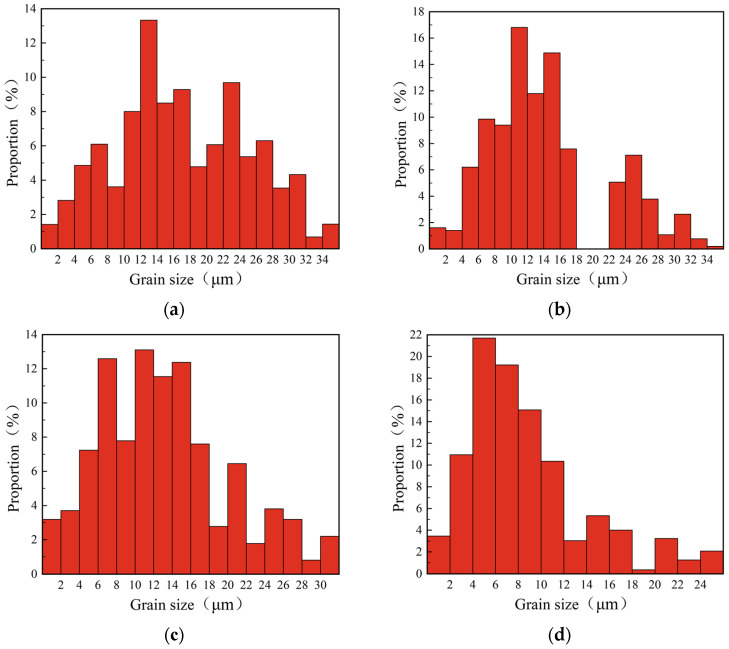
Grain size distribution in the metamorphic layer of GH4169 cutting surface under different flank wears: (**a**) VB = 0 mm; (**b**) VB = 0.1 mm; (**c**) VB = 0.2 mm; (**d**) VB = 0.3 mm.

Figure 17 shows that the flank wear width was 0 mm, 0.1 mm, 0.2 mm, and 0.3 mm, and the proportion of grains with a size less than 16 μm in the cutting metamorphic layer was 44.40%, 68.92%, 64.64%, and 74.71%, correspondingly. That is to say as the width of the flank wear increased, the degree of grain refinement in the metamorphic layer of the cutting surface gradually increased. Rachid M’Saoubi [32] observed obvious recrystallization and grain refinement in the plastic deformation layer caused by worn tool using TEM.

## 6. Influence of Flank Wear on the Grain Boundary Misorientation

**Figure 18:**
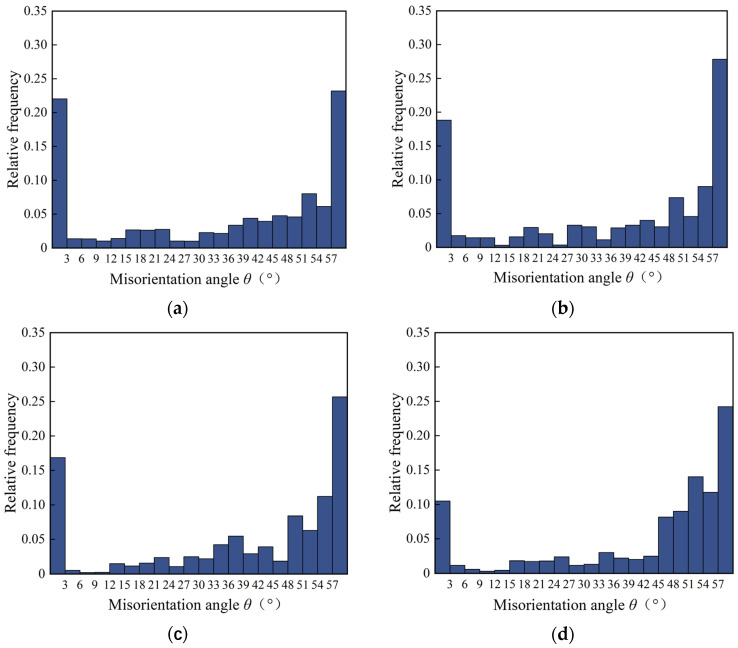
Distribution of grain boundary misorientation in the metamorphic layer of the GH4169 cutting surface under different flank wears: (**a**) VB = 0 mm; (**b**) VB = 0.1 mm; (**c**) VB = 0.2 mm; (**d**) VB = 0.3 mm.

Figure 18 shows the statistics for the grain boundary misorientation distribution under different flank wear conditions. When the flank wear width was 0 mm, 0.1 mm, 0.2 mm, and 0.3 mm, the average grain boundary misorientation in the cutting metamorphic layer was 34.47°, 36.26°, 38.84°, and 43.93°, which means that the average grain boundary misorientation in the cutting metamorphic layer increased as the flank wear was exacerbated. Hines [33] reported in previous research that a high strain and strain rate caused the twin layer to become a refined subgrain, and with the increase of cutting deformation, the subgrain underwent rotation adaptation deformation, which led to a greater difference in orientation and finally formed recrystallization grains.

**Figure 19:**
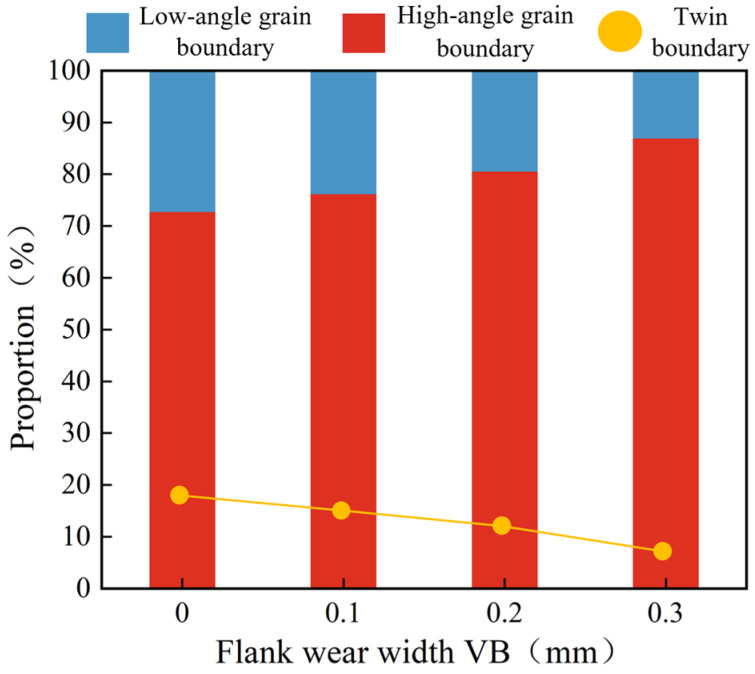
Proportion of high-low angle grain boundaries and twin grain boundaries under different flank wears.

Figure 19 shows the percentage of high-low angle grain boundaries and twin grain boundaries under the different flank wear conditions. The percentage of high-angle grain boundaries in the cutting metamorphic layer was 72.84%, 76.26%, 80.67%, and 87.01% when the flank wear width was 0 mm, 0.1 mm, 0.2 mm, and 0.3 mm, while the proportion of twin grain boundaries was 18.43%, 15.56%, 12.82%, and 8.65%, correspondingly. That is, increasing flank wear width caused an increase in the high-angle grain boundary content and a decrease in twin grain boundary content. Driven by severe plastic deformation, twin grain boundaries were transformed into large-angle grain boundaries [34].

## 7. Conclusions

Combining simulation and experiments, this paper investigated the effect of flank wear on the microstructure characteristics of the metamorphic layer of the cutting surface while cutting superalloy GH4169 under high-pressure cooling. In summary, the following three conclusions were reached:

1. Through simulation of the turning process, it was discovered that increasing the flank wear width improved the cutting force, cutting temperature, plastic strain, and strain rate, and the plastic deformation layer was thicker. In the cutting experiment, when VB was 0.3 mm, the *F_x_* increased by 203 N, the tangential *F_y_* increased by 277 N, and the radial *F_z_* increased by 237 N. In addition, the relative error between the experimental results and the simulation results of cutting force was less than 15%, which proved the reliability of the simulation.
2. In the metamorphic layer, the grains were broken, the grain boundaries were blurred, and there were deformation twins. With the increase of flank wear, the thickness of the metamorphic layer increased from 4.5 μm to 8.7 μm.
3. The average grain size of the cutting surface became larger through the sectional depth of the workpiece. When the flank wear width was 0 mm, 0.1 mm, 0.2 mm, and 0.3 mm, the average grain size in the cutting metamorphic layer was 16.22 μm, 14.50 μm, 11.82 μm, and 6.46 μm; the proportion of grains with a size less than 16 μm was 44.40%, 68.92%, 64.64%, and 74.71%; the average grain boundary misorientation was 34.47°, 36.26°, 38.84°, and 43.93°; the percentage of high-angle grain boundaries was 72.84%, 76.26%, 80.67%, and 87.01%; and the proportion of twin grain boundaries was 18.43%, 15.56%, 12.82%, and 8.65%, respectively.

## Figures and Tables

**Table 1:** Simulation parameters.

Workpiece Height (mm)	Workpiece Length (mm)	Cutting Edge Radius (mm)	Rake Angle	Relief Angle	Rake Length (mm)	Relief Length (mm)
5	10	0.8	6°	−6°	3	3
**Minimum element size** **(mm)**	**Maximum element size** **(mm)**	**Mesh Grading**	**Cutting speed (m/min)**	**Feed rate (mm/r)**	**Cutting depth** **(mm)**	**Coolant pressure** **(bar)**
0.1	1	0.5	160	0.15	0.3	50

**Table 2:** Chemical composition of the superalloy GH4169 (mass fraction, %).

Ni	Cr	Nb	Mo	Ti	Al	Si	Co	Mn	Cu	C	Fe
53.22	19.08	5.13	3.17	1.12	0.43	0.13	0.11	0.05	0.05	0.035	other

**Table 3:** Physical properties of the superalloy GH4169 (20 °C).

Hardness *HB* (N/mm^2^)	Impact Toughness *ak* (MJ/m^2^)	Density *ρ* (kg/m^3^)	Shrinkage Ratio *ψ* (%)	Tensile Strength *σ_b_* (MPa)	Yield Strength *σ*_0.2_ (MPa)	Elongation *δ_s_* (%)
410~420	348	8438	45.5	1376	1152	19

**Table 4:** Geometric parameters of the PCBN inserts.

Insert Shape	Tool Tip Angle	Tool Edge Radius (mm)	Effective Cutting Edge Length (mm)	Diameter of Inscribed Circle (mm)	Clearance Angle	Thickness (mm)
Rhombus	80°	0.8	3	12.7	0°	4.76

**Table 5:** Experiment parameter design.

Serial Number	Flank Wear Width VB (mm)	Cutting Parameters
1	0	*v_c_* = 160 m/min, *f* = 0.15 mm/r, *a_p_* = 0.3 mm, *P* = 50 bar
2	0.1	
3	0.2	
4	0.3	

**Table 6:** Comparison of cutting force data.

Serial Number	Flank Wear Width VB (mm)	Experimental Results			Simulation Results *F_y_* (N)	Relative Error (%)
		Axial Force *F_x_* (N)	Tangential Force *F_y_* (N)	Radial Force *F_z_* (N)		
1	0	128.3	201.2	96.36	225.89	12.27
2	0.1	165.1	247.22	136.56	269.14	8.89
3	0.2	190.65	278.5	157.38	306.92	10.21
4	0.3	331.18	478.69	333.38	443.52	−7.35

## Data Availability

Not applicable.
